# Rhythmic Sensory Stimulation and Music-Based Interventions in Focal Epilepsy: Clinical Evidence, Mechanistic Rationale, and Digital Perspectives—A Narrative Review

**DOI:** 10.3390/jcm15010288

**Published:** 2025-12-30

**Authors:** Ekaterina Andreevna Narodova

**Affiliations:** Department of Neurology, Prof. V.F. Voyno-Yasenetsky Krasnoyarsk State Medical University, 660022 Krasnoyarsk, Russia; katya_n2001@mail.ru; Tel.: +7-902-924-89-95

**Keywords:** focal epilepsy, drug-resistant epilepsy, rhythmic sensory stimulation, music therapy, neurologic music therapy, neural entrainment, neuromodulation, digital health, self-management

## Abstract

Background: Rhythmic sensory stimulation, including structured musical interventions, has gained renewed interest as a non-pharmacological strategy that may modulate cortical excitability and network stability in focal epilepsy. Although several small studies have reported changes in seizure frequency or epileptiform activity during rhythmic or music exposure, the underlying mechanisms and translational relevance remain insufficiently synthesized. Objective: This narrative review summarizes clinical evidence on music-based and rhythmic sensory interventions in focal epilepsy, outlines plausible neurophysiological mechanisms related to neural entrainment and large-scale network regulation, and discusses emerging opportunities for digital delivery of rhythmic protocols in everyday self-management. Methods: A structured search of recent clinical, neurophysiological, and rehabilitation literature was performed with emphasis on rhythmic auditory, tactile, and multimodal stimulation in epilepsy or related conditions. Additional theoretical and translational sources addressing oscillatory dynamics, entrainment, timing networks, and patient-centered digital tools were reviewed to establish a mechanistic framework. Results: Existing studies—although limited by small cohorts and heterogeneous methodology—suggest that certain rhythmic structures, including specific musical compositions, may transiently modulate cortical synchronization, reduce epileptiform discharges, or alleviate seizure-related symptoms in selected patients. Evidence from neurologic music therapy and rhythmic stimulation in other neurological disorders further supports the concept that externally delivered rhythms can influence timing networks, attentional control, and interhemispheric coordination. Advances in mobile health platforms enable structured rhythmic exercises to be delivered and monitored in real-world settings. Conclusions: Music-based and rhythmic sensory interventions represent a promising but underexplored adjunctive approach for focal epilepsy. Their effectiveness likely depends on individual network characteristics and on the structure of the applied rhythm. Digital integration may enhance personalization and adherence. Rigorous clinical trials and mechanistic studies are required to define optimal parameters, identify responders, and clarify the role of rhythmic stimulation within modern epilepsy care.

## 1. Introduction

Focal epilepsy remains a major cause of neurological disability worldwide, and up to one third of patients develop drug-resistant epilepsy (DRE) despite adequate trials of appropriately chosen antiseizure medications (ASMs) [1,2]. The International League Against Epilepsy (ILAE) defines DRE as failure of two tolerated and correctly used ASM schedules to achieve sustained seizure freedom, underscoring the need for alternative and adjunctive treatment strategies beyond conventional pharmacotherapy [1]. In this context, neuromodulatory approaches—including resective surgery, vagus nerve stimulation, deep brain stimulation, and responsive neurostimulation—have gained importance but remain inaccessible or unsuitable for many patients due to anatomical, cognitive, or resource-related constraints [2].

Non-pharmacological, non-invasive interventions are therefore attracting renewed interest as potential adjuncts in DRE, particularly those capable of modulating large-scale brain networks without surgery or systemic drug exposure. Among these, rhythmic sensory stimulation and music-based interventions occupy a specific niche. A growing body of clinical reports suggests that exposure to particular musical structures—most notably Mozart’s Sonata K.448—may reduce epileptiform discharges and, in some cases, seizure frequency in people with difficult-to-treat epilepsy [3]. However, despite a recent systematic review indicating several level-1 studies supporting the so-called “Mozart effect”, the overall evidence base remains heterogeneous, with small samples, variable protocols, and inconsistent long-term outcomes [3]. As a result, music-based interventions are still perceived as experimental rather than integrated elements of epilepsy care.

In parallel, neurologic music therapy has developed into a structured rehabilitation discipline across other neurological conditions, including stroke, Parkinson’s disease, and neurodegenerative disorders [4,5]. In these populations, rhythm-based protocols—such as rhythmic auditory stimulation for gait, patterned sensory enhancement, and melodic intonation therapy—have been shown to influence motor timing, attention, and cognitive–emotional regulation, supporting the broader concept that externally delivered rhythms can shape large-scale neural dynamics [4,5]. Translating these principles to epilepsy raises the hypothesis that carefully designed rhythmic sensory inputs might transiently stabilize cortical networks, improve top-down control, or interfere with pathways that promote secondary generalization.

From a systems-neuroscience perspective, rhythmic stimulation can be conceptualized within the framework of neural entrainment, in which periodic external input aligns ongoing oscillatory activity and alters effective connectivity between key hubs. Recent theoretical work in focal epilepsy has proposed that multisensory low-frequency rhythms may modulate prefrontal timing networks and interhemispheric coordination, thereby influencing network stability during the vulnerable transition from focal onset to bilateral tonic–clonic seizures [6,7]. Within this framework, the structure of the applied rhythm (tempo, regularity, predictability, modality) and the individual’s intrinsic timing characteristics may critically determine whether stimulation facilitates stabilization or inadvertently promotes hypersynchrony.

Parallel advances in digital epileptology open additional translational opportunities. Mobile and wearable tools for seizure monitoring and self-management are increasingly used to support documentation, medication adherence, and communication with clinicians [6]. Narrative and systematic reviews indicate that digital health solutions are gradually evolving from passive diaries toward interactive platforms that integrate prediction algorithms, behavioral support, and, in some cases, prototype neuromodulatory modules [6]. Integrating rhythmic sensory protocols—such as tailored musical playlists, metronome-like cues, or multimodal rhythmic exercises—into these digital ecosystems could enable safe, patient-initiated delivery of structured rhythms in real-world settings, with continuous monitoring of seizure dynamics and user engagement.

Given these developments, there is a need for a focused narrative synthesis that brings together clinical data on music-based and rhythmic sensory interventions in epilepsy, mechanistic insights from neurologic music therapy and neural entrainment research, and emerging digital implementation pathways. Although rhythmic and music-based interventions have been explored in epilepsy for several decades, the available evidence remains fragmented, methodologically heterogeneous, and rarely discussed within an integrated systems-neuroscience and digital health framework. This relative lack of synthesis represents an important gap, particularly for focal epilepsy. The present review aims (I) to summarize existing clinical and experimental evidence on rhythmic auditory and multimodal stimulation in focal epilepsy, with particular attention to structured musical interventions; (II) to outline plausible neurophysiological mechanisms linking rhythmic input, timing networks, and large-scale epileptic dynamics; and (III) to discuss how digital platforms, including mobile applications, might operationalize individualized rhythmic protocols as part of everyday self-management.

## 2. Methods

This work was conducted as a narrative review with the aim of synthesizing clinical, mechanistic, and translational perspectives on rhythmic sensory and music-based interventions in focal epilepsy. A structured but non-systematic literature search was initially performed in January 2025 and updated in November 2025 using PubMed, Scopus, and Web of Science. No date restrictions were applied initially; however, emphasis was placed on studies published within the past 15–20 years due to the evolution of modern neuromodulation concepts and digital health tools.

Clinical studies were included if they reported effects of music, rhythmic auditory, or multimodal sensory stimulation on seizure frequency, epileptiform activity, behavioral symptoms, or patient-reported outcomes in individuals with focal or mixed-type epilepsy. Both randomized and non-randomized designs were eligible. Case reports were considered when they illustrated mechanistic or translational relevance. Studies focusing exclusively on photosensitive epilepsy, purely cognitive music training, or non-rhythmic auditory exposures were excluded unless they provided mechanistic insights relevant to rhythmic interventions.

To build a mechanistic framework, the literature from adjacent fields was incorporated when rhythmic stimulation demonstrated behavioral or neurophysiological effects, including neurologic music therapy, rehabilitation neuroscience, and research on neural entrainment and oscillatory network regulation. Digital health sources were reviewed to contextualize opportunities for remote delivery, personalization, and integration of rhythmic protocols into everyday self-management.

Because the evidence base is heterogeneous and often limited by small sample sizes, no quantitative synthesis or pooled effect estimation was attempted. Instead, findings were analyzed qualitatively, with attention to methodological strengths and limitations and to convergence between clinical outcomes, theoretical models, and emerging digital approaches. The goal of this approach was to map the landscape of rhythmic sensory interventions in focal epilepsy, identify conceptual links across disciplines, and outline priorities for future research.

Although this review was not designed as a formal systematic review, the literature search was conducted in a structured and transparent manner, as detailed in Supplementary File S1. Due to substantial heterogeneity in study designs, populations, interventions, and outcome measures, as well as the limited number of epilepsy-specific studies, a PRISMA-compliant systematic review or meta-analysis was not feasible. Therefore, a narrative synthesis was intentionally chosen to integrate clinical observations with mechanistic insights and digital perspectives. The full search strings and adaptations across databases are provided in Supplementary File S1.

During the preparation of this manuscript, the author used ChatGPT (GPT-5.1) for assistance with language polishing and structuring of the text. After using this tool, the author carefully checked and edited the content and takes full responsibility for the final version of the manuscript.

## 3. Results

### 3.1. Clinical Evidence on Music-Based and Rhythmic Sensory Interventions in Epilepsy

Clinical research on music-based and rhythmic sensory interventions in focal epilepsy is limited but evolving. Most work has focused on listening to Mozart’s Sonata for Two Pianos in D Major (K.448) as a non-pharmacological adjunct to conventional treatment, often referred to as the Mozart effect. Several clinical studies have investigated the influence of K.448 on epileptiform discharges and seizure frequency.

In observational and experimental protocols involving children with epilepsy, listening to Mozart K.448 has been associated with reductions in epileptiform discharges and seizure recurrence. For example, pediatric studies demonstrated that daily listening to Mozart K.448 before sleep over several months reduced seizure recurrence rates and epileptiform discharges when compared to control conditions, suggesting a potential therapeutic effect in early seizure management [3]. Similar findings were reported in children with persistent epileptiform activity, where K.448 exposure significantly decreased interictal epileptiform discharges on EEG recordings [3].

Adult epilepsy populations have also been investigated. Randomized crossover trials reported reductions in seizure frequency during daily exposure to Mozart K.448 compared to control auditory stimuli lacking comparable rhythmic structure, highlighting a potential role of rhythmic properties in modulating seizure burden [3]. Randomized controlled findings further indicate that listening to K.448 may be associated with decreased seizure counts compared to non-rhythmic auditory control stimuli, although the precise mechanisms remain unclear [3].

Overall, these studies suggest that certain musical structures may influence epileptiform activity and clinical outcomes in subgroups of patients. However, the evidence base is characterized by small sample sizes, heterogeneous study designs, varying control conditions, and short follow-up periods. Meta-analytic syntheses report modest or inconsistent effects across studies, indicating that the evidential value of the Mozart effect in epilepsy requires cautious interpretation and further replication [3]. Broader analyses and secondary reviews support the notion that music as a therapeutic modality can influence neural activity; however, the specificity of effects for epilepsy remains an area of active investigation, with both positive and null findings reported [3]. To improve traceability of the summarized protocols, Table 1 now provides key references for each listed intervention. A summary of clinical observations is provided in Table 1.

### 3.2. Evidence from Neurologic Music Therapy and Other Neurological Disorders

Neurologic music therapy (NMT) has evolved into an evidence-based rehabilitation discipline demonstrating that structured rhythmic and musical interventions can modulate large-scale neural networks across multiple neurological conditions. Although epilepsy-specific evidence remains limited, findings from stroke, Parkinson’s disease, traumatic brain injury, and neurodegenerative disorders offer mechanistic insights relevant to rhythmic modulation of cortical dynamics.

In stroke rehabilitation, rhythmic auditory stimulation (RAS) has consistently been shown to improve gait velocity, stride symmetry, and motor coordination by entraining movement to an external auditory rhythm. Numerous clinical trials and meta-analyses demonstrate that RAS enhances motor timing, increases sensorimotor coupling, and facilitates recruitment of preserved cortical–subcortical circuits [4]. These effects suggest that rhythmic cues can regulate temporal predictability and reduce motor variability—mechanisms conceptually relevant to stabilizing neuronal activity in epilepsy, where timing dysregulation and impaired top-down control are frequently observed.

In Parkinson’s disease, rhythmic auditory cues improve bradykinesia, gait freezing, and motor initiation, with several studies reporting measurable changes in cortical oscillations and synchronization patterns during rhythmic training. A recent systematic review and meta-analysis confirmed that NMT protocols—including RAS, patterned sensory enhancement, and tempo-based movement training—produce significant benefits in both motor and non-motor symptoms, supporting the view that rhythm engages distributed timing networks and modulates beta-band activity implicated in motor control [5]. These findings emphasize rhythm’s capacity to reorganize dysfunctional networks, even in disorders characterized by impaired basal ganglia circuitry.

Beyond motor systems, rhythmic and melodic interventions have demonstrated cognitive and emotional effects. Studies in traumatic brain injury and dementia populations show improvements in attention, verbal fluency, executive control, and affect regulation following structured musical training. Mechanistically, these effects have been linked to enhanced interhemispheric coordination, increased functional connectivity, and strengthened engagement of prefrontal and temporoparietal networks [4]. Such mechanisms overlap with theoretical models of network instability in focal epilepsy, particularly regarding disrupted prefrontal timing, impaired inhibitory control, and heightened susceptibility to hypersynchrony.

Collectively, evidence from neurologic music therapy indicates that rhythmic and musical stimuli can systematically influence motor, cognitive, and affective functions through modulation of timing networks, oscillatory synchronization, and large-scale connectivity. Although direct translation to epilepsy requires caution, these findings support the broader hypothesis that well-structured rhythms may stabilize vulnerable neural circuits or alter excitability states—providing a plausible mechanistic rationale for exploring rhythmic sensory stimulation as an adjunctive approach in focal epilepsy.

### 3.3. Mechanistic Rationale: Neural Entrainment, Oscillations, and Network Stability

Rhythmic sensory stimulation interacts with the brain’s intrinsic tendency to organize activity into oscillatory patterns. Neural oscillations provide a temporal framework for coordinating communication between distributed regions, and their phase alignment can facilitate or impede information flow. Experimental work in humans demonstrates that non-invasive rhythmic stimulation—such as periodic visual flashes, auditory clicks, or transcranial magnetic stimulation—can entrain ongoing oscillations, shifting their phase and amplitude in a frequency-specific manner [8,9]. Within this framework, external rhythms act as a temporal scaffold that biases when neuronal populations are more likely to fire and synchronize, thereby shaping perception, attention, and behavior [8,9,10].

**Figure 1:**
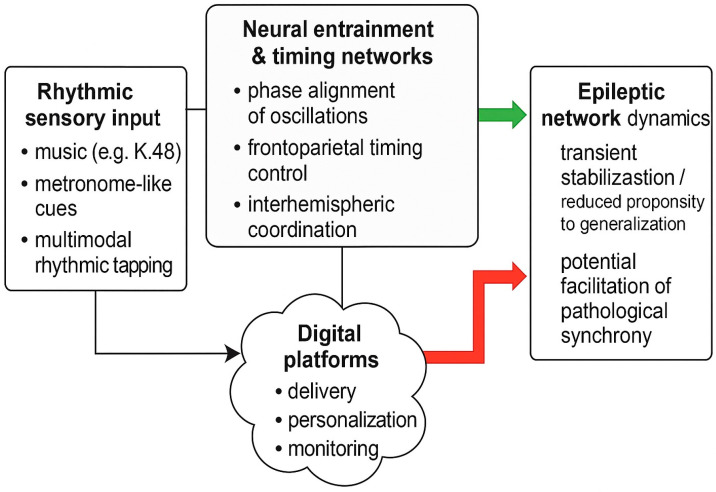
Conceptual framework linking rhythmic sensory input, neural entrainment, and epileptic network dynamics in focal epilepsy. Rhythmic sensory stimulation (**left**), including structured music, metronome-like cues, and multimodal rhythmic tapping, interacts with timing networks to influence phase alignment of oscillations, frontoparietal timing control, and interhemispheric coordination (**center**). These processes can modulate epileptic network dynamics (**right**), potentially leading either to transient stabilization and reduced propensity to secondary generalization (green arrow) or, under unfavorable conditions, to facilitation of pathological synchrony (red arrow). Digital platforms (**bottom**) provide a medium for delivering, personalizing, and monitoring rhythmic protocols in real-world settings.

Entrainment and network resonance have been proposed as core mechanisms of top-down attentional control. A conceptual overview is presented in Figure 1. Low-frequency rhythms in frontoparietal networks appear to sample sensory information in a cyclic manner, with behavioral performance fluctuating at theta–alpha rates in tasks requiring sustained attention [10]. When external stimulation matches the intrinsic timescales of these networks, resonance phenomena can emerge, amplifying the impact of rhythmic inputs on large-scale connectivity [10]. Conversely, mismatched or irregular rhythms may fail to drive stable entrainment or could introduce additional temporal noise. These principles suggest that the clinical impact of rhythmic interventions in epilepsy may depend critically on how closely the applied rhythm aligns with an individual’s intrinsic oscillatory dynamics and network susceptibilities.

Epileptic networks are characterized by complex, time-varying patterns of synchronization and desynchronization rather than a simple state of “hyper-synchrony”. Experimental and computational studies show that seizures often involve an initial phase of local desynchronization, followed by evolving patterns of regional coupling and, in some cases, highly synchronized activity toward seizure termination [11]. This dynamic interplay reflects underlying changes in excitatory–inhibitory balance, network topology, and the recruitment of long-range connections. From a mechanistic standpoint, rhythmic sensory stimulation could influence these trajectories by biasing phase relationships within and between critical hubs, including prefrontal and limbic regions implicated in seizure propagation and secondary generalization.

Recent theoretical work in focal epilepsy has integrated these ideas into a systems-level framework, proposing that multisensory low-frequency rhythms may modulate prefrontal timing networks and interhemispheric coordination, thereby affecting network stability during transitions from focal onset to bilateral tonic–clonic seizures [7]. In this view, prefrontal circuits act as a temporal control hub whose ability to maintain balanced, flexible connectivity is vulnerable to perturbations in internal timekeeping and oscillatory coherence. Exogenous rhythmic stimulation—delivered through auditory, tactile, or combined channels—could, at least transiently, reinforce more stable timing patterns and reduce the likelihood that vulnerable networks drift into configurations favoring seizure generalization.

At the same time, the bidirectional nature of synchronization in epilepsy underscores a key caution: externally imposed rhythms are not inherently protective. Depending on frequency, phase relationships, and network state, rhythmic inputs might either stabilize dynamics or inadvertently facilitate pathological synchronization. Mechanistic models therefore support a nuanced perspective in which rhythmic interventions are viewed not as universally beneficial, but as tools whose effects depend on careful parameter selection and individualized understanding of network behavior. This rationale underpins the need for systematic exploration of rhythm structure, sensory modality, and timing relative to seizure dynamics when designing future clinical protocols in focal epilepsy. Key mechanistic principles and their implications for focal epilepsy are summarized in Table 2.

### 3.4. Digital Perspectives: From Rhythmic Protocols to Self-Management Platforms

Digital health technologies are reshaping how individuals with epilepsy monitor symptoms, manage medications, and interact with clinical teams. Modern mobile applications, wearable sensors, and cloud-based platforms increasingly integrate multimodal information—behavioral logs, physiological signals, sleep metrics, and environmental factors—and support patient-driven self-management. Recent reviews emphasize that digital tools have progressed from simple seizure diaries to more sophisticated systems capable of real-time tracking, decision support, and individualized behavioral interventions [6,12].

Within this evolving ecosystem, rhythmic sensory stimulation represents a potential functional extension of digital self-management. Unlike traditional neuromodulation devices, mobile platforms can deliver rhythmic auditory, tactile, or multimodal cues in a flexible, user-controlled manner. Such implementation enables patients to access structured rhythmic exercises during vulnerable periods, integrate them into daily routines (e.g., pre-sleep, stress-related triggers), and receive continuous feedback on adherence or symptom patterns. Early work in digital neurology highlights that personalized behavioral modules can enhance engagement and improve management outcomes in chronic neurological conditions [12,13], suggesting a feasible path for incorporating rhythmic protocols into patient-centered care.

Digital delivery also aligns with mechanistic considerations. If the effects of rhythmic stimulation depend on timing, regularity, and individual oscillatory traits, mobile systems offer the possibility of fine-tuning tempo, modality, and session duration based on user preference or clinical profile. Over time, data-driven personalization—drawing on seizure logs, physiological markers, or cognitive performance—could help identify responders and optimize stimulation parameters. Importantly, such approaches remain exploratory and require careful clinical validation to ensure safety, feasibility, and avoidance of inadvertent overstimulation.

A practical example of this concept is the integration of rhythmic elements into digital self-management tools designed for focal epilepsy. Prototype platforms that combine seizure logging, medication reminders, and structured rhythmic exercises illustrate how rhythmic modulation and behavioral support can coexist within a single application framework [6]. While these implementations do not constitute therapeutic neuromodulation in the traditional sense, they demonstrate a translational pathway for delivering rhythmic cues in real-world environments, supporting adherence and potentially stabilizing daily routines associated with seizure thresholds.

Telehealth infrastructures further expand these possibilities, allowing clinicians to remotely monitor patient-generated data and adjust behavioral recommendations. Remote monitoring programs in epilepsy have already shown improved communication, earlier identification of seizure worsening, and enhanced patient satisfaction [13]. Embedding rhythmic protocols into such systems could facilitate feedback-driven iteration and support hybrid models of care that combine behavioral, digital, and pharmacological components.

Overall, digital platforms offer a versatile medium for implementing rhythmic sensory stimulation in a personalized and scalable manner. Their value lies not in replacing established therapies but in complementing existing management frameworks, enabling structured experimentation, and accelerating mechanistic and clinical discovery in the field of rhythmic interventions for focal epilepsy.

## 4. Discussion

The present narrative review synthesizes evidence from clinical, mechanistic, and digital domains to outline the potential role of rhythmic sensory and music-based interventions in focal epilepsy. Although the body of clinical data remains limited and heterogeneous, several consistent themes emerge that provide a coherent rationale for further exploration.

### 4.1. Clinical Observations and Their Constraints

Studies evaluating music—particularly Mozart’s Sonata K.448—suggest transient reductions in epileptiform discharges and seizure frequency in selected individuals with epilepsy [3]. These effects appear across pediatric and adult populations, though with considerable variability in study design, control conditions, and outcome measures [3]. Importantly, most available studies are characterized by small sample sizes, short follow-up periods, and limited blinding, restricting the strength of inferences that can be drawn. Importantly, long-term outcomes of rhythmic and music-based interventions in epilepsy remain largely unexplored. Most available studies report effects over weeks to a few months, and data beyond 6–12 months are scarce or absent. Consequently, the durability of observed reductions in epileptiform activity or seizure frequency cannot be reliably determined at present. Nevertheless, the recurrence of beneficial patterns across independent cohorts indicates that rhythmic or structural properties of music may modulate cortical activity in clinically meaningful ways. However, such findings must be interpreted cautiously, as publication bias, heterogeneous patient profiles, and non-standardized protocols may have contributed to inflated estimates of efficacy.

### 4.2. Insights from Neurologic Music Therapy and Adjacent Fields

Evidence from neurologic music therapy reinforces the concept that structured rhythmic cues can influence distributed timing networks, motor control, cognitive processing, and emotional regulation [4,5]. These effects are mechanistically linked to entrainment and resonance phenomena, in which rhythmic input reshapes oscillatory dynamics, synchrony, and connectivity. Although derived from stroke, Parkinson’s disease, and other neurological conditions, such findings illustrate that externally delivered rhythms can modulate functional networks beyond primary sensory pathways. This broader context strengthens the rationale for applying rhythmic protocols in epilepsy, where disruptions in timing, connectivity, and large-scale coordination are integral components of seizure vulnerability and propagation.

### 4.3. Mechanistic Plausibility Grounded in Oscillatory Neuroscience

Mechanistic evidence supports the notion that rhythmic sensory stimulation may influence epileptic networks through entrainment of intrinsic oscillations and phase alignment across cortical regions [8,9,10]. Neural entrainment can enhance or suppress communication between neuronal populations depending on frequency, timing, and network state. Computational and physiological models of epilepsy further indicate that seizure evolution involves dynamic fluctuations between synchronization and desynchronization rather than a uniform state of hypersynchrony [11]. Within this framework, rhythmic input could theoretically stabilize vulnerable circuits by reinforcing more regular phase relationships or improving top-down modulation by prefrontal timing networks—an idea captured in recent systems-level hypotheses of focal epilepsy [7]. At the same time, the bidirectional nature of synchronization emphasizes the importance of careful parameter selection, as inappropriate rhythmic inputs may inadvertently amplify pathological coupling.

### 4.4. Digital Integration as a Translational Pathway

Digital health technologies offer a promising platform for operationalizing rhythmic sensory interventions within everyday self-management. Modern mobile applications and telemonitoring systems enable remote delivery, personalization, and iterative adjustment of behavioral modules in epilepsy care [6,12,13]. Embedding rhythmic exercises into these systems could allow patients to access structured interventions during periods of heightened vulnerability, track adherence, and document subjective or objective responses. Moreover, digital platforms may facilitate identification of responders, enable real-world data collection, and support hybrid approaches that combine rhythmic stimulation with conventional pharmacological and neuromodulatory therapies.

However, the digital pathway remains exploratory. No current application provides validated therapeutic rhythmic stimulation for epilepsy, and safety considerations—including risk of overstimulation, discomfort, or unintended triggering of symptoms—require thorough clinical evaluation. Nonetheless, early integration of rhythmic components into self-management applications [6] illustrates a feasible and patient-centered route for controlled, scalable implementation. These considerations align with recent systems-level hypotheses proposing that multisensory low-frequency entrainment may support prefrontal network stability in focal epilepsy [7]. In parallel, digital self-management tools for epilepsy increasingly incorporate structured behavioral components [6], suggesting that rhythmic sensory stimulation could be operationalized within the same conceptual framework.

### 4.5. Limitations and Future Directions

Across domains, several limitations should be acknowledged. First, heterogeneity in clinical protocols prevents meaningful comparison of rhythmic interventions and precludes meta-analytic synthesis. Second, mechanistic studies often rely on non-epilepsy populations, limiting direct translational inference. Third, digital approaches must balance personalization with safety, ensuring that data-driven adaptations do not introduce unintended neural consequences. Future research should focus on the following:

(1) Standardized rhythmic protocols with defined tempo, structure, and duration;
(2) Mechanistic studies integrating EEG/MEG markers of entrainment and network stability;
(3) Identification of individual predictors of response;
(4) Controlled digital delivery frameworks enabling safe at-home implementation; and
(5) Robust randomized clinical trials evaluating rhythmic stimulation as an adjunct to standard epilepsy therapies.

### 4.6. Overall Interpretation

In summary, the available evidence supports a cautious but scientifically grounded view that rhythmic sensory and music-based interventions may influence epileptic network dynamics. The convergence of clinical observations, mechanistic plausibility, and digital feasibility suggests a promising direction for future investigation. Rather than positioning rhythmic stimulation as an established therapy, the current synthesis highlights its potential as an adjunctive, personalized tool whose efficacy depends on individual network properties and thoughtfully designed protocols. Continued research across clinical, computational, and digital domains will be essential to determine the true therapeutic value of rhythmic approaches in focal epilepsy.

## 5. Conclusions

This narrative review highlights emerging evidence that rhythmic sensory and music-based interventions may modulate cortical dynamics in focal epilepsy. Although preliminary findings suggest that structured rhythmic inputs—particularly specific musical compositions—can influence epileptiform activity and seizure-related outcomes in selected patients, current studies are limited by small samples, heterogeneous protocols, and short follow-up periods. Mechanistic insights from oscillatory neuroscience and neurologic music therapy provide a plausible framework in which rhythmic stimulation may transiently stabilize vulnerable networks through entrainment, resonance, and enhanced timing control. Digital health technologies offer an opportunity to integrate such interventions into patient-centered self-management, enabling personalized delivery, real-world monitoring, and iterative refinement.

At present, rhythmic sensory stimulation should be regarded as an adjunctive, exploratory approach rather than an established therapeutic modality. Clarifying its clinical value will require standardized rhythmic protocols, rigorous mechanistic evaluation, and well-designed randomized trials. Continued integration of clinical, computational, and digital perspectives will be essential for determining whether rhythmic interventions can meaningfully contribute to modern care pathways for individuals with focal epilepsy.

## Figures and Tables

**Table 1:** Clinical observations on music-based and rhythmic sensory interventions in epilepsy.

Population	Intervention	Control Condition	Duration	Outcomes Assessed	Main Findings	Key Limitations	Key References
Children with focal or mixed epilepsy	Daily listening to Mozart K.448 (often pre-sleep)	Silence or non-structured music	Weeks–months	Seizure frequency; epileptiform discharges (IEDs)	↓ IEDs; ↓ seizure recurrence in several cohorts	Small N; no blinding; heterogeneous protocols	[3]
Children with persistent IEDs	Repeated exposure to K.448 during EEG	Non-rhythmic auditory control	Single session to repeated sessions	IED count; EEG morphology	Clear short-term ↓ in IEDs	Short-term design only	[3]
Adults with focal epilepsy	Daily listening to K.448 (home-based)	Non-rhythmic auditory stimulus	1–3 months	Monthly seizure frequency	↓ seizures during K.448 exposure periods	Cross-over design; no sham control	[3]
Mixed adult/pediatric DRE populations	Music exposure protocols varying by tempo and structure	Silence; unrelated music	Variable	Seizure logs; IEDs; patient-reported symptoms	Several studies report beneficial effects, but not all; effects inconsistent	Heterogeneous designs; publication bias possible	[3]
Across all groups	Any structured rhythmic music	Multiple	Various	Various	General pattern: rhythmic musical structure may modulate cortical excitability	No consensus on optimal rhythm; long-term (>6–12 months) effects insufficiently studied.	[3]

Abbreviations: IEDs, interictal epileptiform discharges; DRE, drug-resistant epilepsy. Key references indicate the main sources supporting each summarized protocol. The symbol ‘↓’ indicates a reduction or decrease in the reported outcome (e.g., reduced seizure frequency or interictal epileptiform discharges).

**Table 2:** Mechanistic principles of rhythmic sensory stimulation and potential relevance for focal epilepsy.

Mechanistic Concept	Supporting Evidence (Field + Ref)	Neurophysiological Implications	Potential Relevance for Epilepsy
Neural entrainment	Cognitive neuroscience [8,9,10]	Phase alignment; modulation of oscillatory amplitude	Biases timing of epileptic hubs; may stabilize transitions
Network resonance	Attention and timing research [9,10]	Amplification when external rhythm matches intrinsic frequency	Enhances impact of rhythmic input when matched to patient-specific dynamics
Frontoparietal timing control	Entrainment studies [8,9,10]	Top-down modulation of temporal prediction and attention	Improves inhibitory control; may counteract destabilizing fluctuations
Interhemispheric coordination	NMT and tract-level findings [4,5]	Increased coherence; improved cross-hemisphere communication	May reduce vulnerability to secondary generalization
Synchronization/desynchronization dynamics	Epilepsy physiology [11]	Seizures involve dynamic shifts, not static hypersynchrony	Rhythmic input may interrupt or reshape propagation patterns
Multimodal integration	NMT and sensory research [4,5]	Stronger entrainment with combined modalities	Potential for more robust stabilization via multisensory input
Digital personalization	Digital health [6,12,13]	Tailored tempo, modality, session timing	Optimizes response; identifies responders; improves adherence

## Data Availability

No new data were created or analyzed in this study. Data sharing is not applicable to this article.

## Supplementary Materials

The following supporting information can be downloaded at: https://www.mdpi.com/article/10.3390/jcm15010288/s1, File S1: Literature Search Strategy; Table S1: Methodological limitations and potential sources of bias in included studies.
