# Supplementary material for: Rhythmic Sensory Stimulation and Music-Based Interventions in Focal Epilepsy: Clinical Evidence, Mechanistic Rationale, and Digital Perspectives—A Narrative Review
Source: J Clin Med. 2025 Dec 30;15(1):288. doi: 10.3390/jcm15010288 (PMC12787044; doi:10.3390/jcm15010288)
Supplement: Supplementary file 1 [file jcm-15-00288-s001.zip › jcm-4067981-supplementary.pdf]

Supplementary File S1. Literature Search Strategy

Search Objective

The aim of the literature search was to identify clinical, mechanistic, and translational studies relevant to rhythmic sensory stimulation, music-based interventions, neural entrainment, and digital self-management in focal epilepsy. This supplementary file outlines the search strategy used to support the narrative synthesis presented in the manuscript.

Databases Searched

The following databases were searched between January and November 2025:

• PubMed/MEDLINE
• Scopus
• Web of Science Core Collection

Additional targeted searches were performed using Google Scholar to identify gray literature and recently published reviews.

Search Terms and Boolean Operators

The search strategy combined terms related to epilepsy, rhythmic stimulation, music-based interventions, neural entrainment, oscillatory dynamics, neurologic music therapy, and digital health. Search terms included the following combinations:

• "epilepsy" OR "focal epilepsy" OR "drug-resistant epilepsy" AND
• "music therapy" OR "Mozart effect" OR "rhythmic stimulation" OR "auditory stimulation" OR "sensory entrainment" AND
• "neural entrainment" OR "oscillatory dynamics" OR "network stability" OR "timing networks" AND
• "digital health" OR "mobile applications" OR "self-management"

Search terms were adapted for each database according to its indexing structure.

Inclusion Criteria

Studies were included if they met any of the following criteria:

• Clinical studies (randomized, non-randomized, observational, crossover, EEG-based) evaluating music or rhythmic sensory stimulation in epilepsy.
• Studies assessing neurologic music therapy or rhythmic interventions in other neurological disorders when mechanistic insights were relevant to epilepsy.

• Experimental or theoretical work on neural entrainment, timing networks, oscillatory synchronization, or multisensory modulation.
• Reviews addressing digital self-management tools in epilepsy.

Exclusion Criteria

Studies were excluded if they met any of the following criteria:
• Non-rhythmic auditory exposure not related to temporal structure.
• Articles focusing exclusively on photosensitive epilepsy without rhythmic stimulation.
• Conference abstracts without accessible full-text versions.
• Non-English publications.

Search Results

This narrative review did not aim to achieve PRISMA-style quantitative synthesis. The search identified a limited set of epilepsy-specific studies on music-based or rhythmic sensory stimulation (fewer than 20), characterized by small sample sizes and methodological heterogeneity. A broader set of studies from neurologic music therapy, cognitive neuroscience, and digital health was incorporated to construct a mechanistic and translational framework.

Rationale for Narrative Synthesis

Due to the heterogeneity of study designs, outcomes, and protocols across the included literature, a formal systematic review or meta-analysis was not feasible. Therefore, a narrative synthesis approach was used to integrate clinical observations with mechanistic insights and emerging digital implementation pathways.

Supplementary Table S1. Methodological characteristics and potential sources of bias in studies on music-based and rhythmic sensory interventions in epilepsy

Study type / design	Sample size (typical range)	Follow-up duration	Blinding	Main potential sources of bias
Observational clinical studies (pediatric epilepsy)	Small (often <50 participants)	Weeks to several months	Not blinded	Selection bias; absence of randomization; caregiver-reported outcomes; short follow-up
EEG-based experimental studies (Mozart K.448 exposure)	Small (often <30 participants)	Single session or short repeated exposure	Not blinded	Short-term assessment only; lack of clinical seizure outcomes; laboratory setting

Study type / design	Sample size (typical range)	Follow-up duration	Blinding	Main potential sources of bias
Randomized crossover trials (adult focal epilepsy)	Small to moderate (≈20–60 participants)	1–3 months per condition	Partial or absent	Carry-over effects; absence of sham control; limited washout periods
Mixed adult/pediatric cohorts with drug-resistant epilepsy	Small, heterogeneous cohorts	Variable; mostly short-term	Not blinded	Clinical heterogeneity; inconsistent outcome measures; publication bias
Music exposure protocols with variable tempo and structure	Variable, often small	Variable	Not blinded	Lack of protocol standardization; unclear dose–response relationship
Narrative syntheses and secondary analyses	Not applicable	Not applicable	Not applicable	Dependence on quality of primary studies; selective reporting in original literature

This table provides a qualitative overview of common methodological limitations and potential sources of bias across the included studies and does not represent a formal systematic risk-of-bias assessment.
